# Incidence and risk factors of postoperative medial shoulder imbalance in Lenke Type 2 adolescent idiopathic scoliosis with lateral shoulder balance

**DOI:** 10.1186/s12891-022-05882-w

**Published:** 2022-11-02

**Authors:** Zhipeng Deng, Liang Wang, Linnan Wang, Xi Yang, Lei Wang, Limin Liu, Yueming Song

**Affiliations:** Department of Orthopedic Surgery and Orthopedic Research Institute, West China Hospital, Sichuan University, No. 37 Guo Xue Xiang, 610041 Chengdu, Sichuan China

**Keywords:** Medial shoulder imbalance, Lateral shoulder balance, Adolescent idiopathic scoliosis

## Abstract

**Background:**

In clinical practice, there are a significant percentage of Lenke 2 AIS patients suffered from medial shoulder imbalance (MSI) despite achieving good lateral shoulder balance (LSB) following surgery. However, there are few studies evaluating the features of the medial shoulder. The objective of this study was to determine the incidence and independent risk factors of MSI with LSB after Lenke 2 AIS corrective surgery.

**Methods:**

One hundred and twenty Lenke 2 AIS patients with LSB at the last follow-up were reviewed from 2009 to 2018. Preoperative, and 3-month and the last postoperative follow-up radiographs were measured using a number of specific measurements. At the last follow-up, patients were divided into medial shoulder balance (MSB) group and the MSI group according to whether the T1 tilt was greater than 3°. A stepwise multiple linear regression analysis was used to examine the independent risk factors for MSI. Scoliosis Research Society (SRS)-30 questionnaire was used to assess clinical outcomes.

**Results:**

Up to 69.2% of patients suffered from MSI with LSB after Lenke Type 2 AIS corrective surgery. Multiple regression showed that postoperative upper instrumented vertebra tilt (UIVt), proximal thoracic curve (PTC), the ratio of PTC and main thoracic curves (PTC/MTC) and T2 vertebra rotation ratio (T2-VR) were significant predictors for MSI (UIVt: b = 0.398, p < 0.001; PTC/MTC: b = 2.085, p < 0.001; PTC: b = 0.155, p < 0.001; T2-VR: b = 3.536, p = 0.008; adjusted R^2^ = 0.711). 72 patients completed the SRS-30 questionnaire survey, and the MSB group were scored the higher (p ≤ 0.001) in self-image domain (4.18 ± 0.43 vs. 3.70 ± 0.35), satisfaction domain (4.39 ± 0.54 vs. 3.95 ± 0.46) and total average (4.31 ± 0.23 vs. 4.11 ± 0.19).

**Conclusion:**

Although the patients with Lenke 2 AIS achieve LSB after corrective surgery, up to 69.2% of them suffered from MSI. Postoperative UIVt, PTC, PTC/MTC and T2-VR were significant predictors for MSI. Sufficient correction of these variables may facilitate the achievement of MSB.

## Background

Adolescent idiopathic scoliosis (AIS) is a complex three-dimensional spinal deformity that is often accompanied by shoulder imbalance. Shoulder imbalance can be classified into two types: lateral and medial shoulder imbalance [1]. Medial shoulder imbalance (MSI) refers clinically to trapezial prominence, which is related to upward tilted proximal ribs and can be evaluated using the T1 tilt angle [1]. Lateral shoulder imbalance (LSI) is reflected by radiographic shoulder balance (RSH) or clavicle angle (Cla-A). Postoperative shoulder imbalance (PSI) from corrective surgery for AIS can affect the patient’s appearance and satisfaction [2]. Therefore, previous studies have been devoted to exploring the incidence and risk factors for PSI to reduce its occurrence. However, almost all studies only used the indices of lateral shoulder as the criteria to assess PSI.

Since stiff proximal thoracic curve (PTC) have a low ability to compensate for the correction gained in the main thoracic curve (MTC), PTC of Lenke 2 AIS patients have been recommended for fusions to reduce the occurrence of PSI [3]. Although the majority of these patients achieved balance of the lateral shoulder after surgery, some of them still complained of asymmetry of the medial shoulder with the bulge clinically on one side. We question whether this phenomenon is common as well as what the causes of this phenomenon are. In our own experience, it seems to be caused by insufficient derotation and correction of the upper proximal thoracic, but to our knowledge, no studies have discussed the phenomenon of MSI with lateral shoulder balance (LSB) after surgery.

Therefore, verifying our conjecture, the aim of this study was to determine the incidence and independent risk factors of MSI with LSB after Lenke 2 AIS corrective surgery.

## Materials and methods

### Design and patient selection

This retrospective cohort study was approved by the Ethics Committee of West China Hospital of Sichuan University (IRB number: 2019 − 852), and informed written consent was obtained from all patients and/or their legal guardians. Data from Type 2 AIS patients who were surgically treated between 2009 and 2018 were analyzed. The inclusion criteria were as follows: (1) age 10 to 20 years at surgery; (2) upper instrumented vertebra (UIV) = T2; (3) one-stage posterior instrumented spinal fusion; (4) a minimum of 2 years after surgery; and (5) LSB at the last follow-up. Patients with incomplete imaging data and an anterior-posterior surgical approach or reoperations were excluded.

### Data collection

Demographic data, including age, Risser sign, and sex, were recorded. Preoperative right and left standing side-bending X-ray films were obtained, and standing posteroanterior and lateral radiographs were obtained before and after surgery. According to the Cobb angle protocol, the degrees of PTC, MTC and thoracolumbar/lumbar curve (TLC) were assessed. At the same time, the PTC/MTC ratio were calculated. The flexibility of these curves was calculated as the preoperative Cobb angle in the standing film minus the side-bending Cobb angle divided by the preoperative Cobb angle. The apical vertebral translation of PTC and MTC (PTC-AVT, MTC-AVT) and coronal balance were also measured. PTC-AVT or MTC-AVT refers to the horizontal distance between the C7 plumb line and the proximal or main thoracic apical vertebra. Coronal balance was defined as the horizontal distance between the C7 plumb line and the central sacral vertical line.

Radiographic shoulder balance (RSH) and T1 tilt angle are commonly used to assess shoulder balance. In this study, the LSB was defined as an RSH less than 10 mm. For the medial shoulder balance, patients were divided into 2 groups based on their T1 tilt angle measured on the last follow-up X-ray films: the MSB group (-3°≤T1 tilt ≤ 3°) and MSI group (T1tilt<;-3° or T1 tilt>3°). These parameters were measured following a published paper [4] (positive is defined as left shoulder up/right shoulder down).

**Fig. 1:**
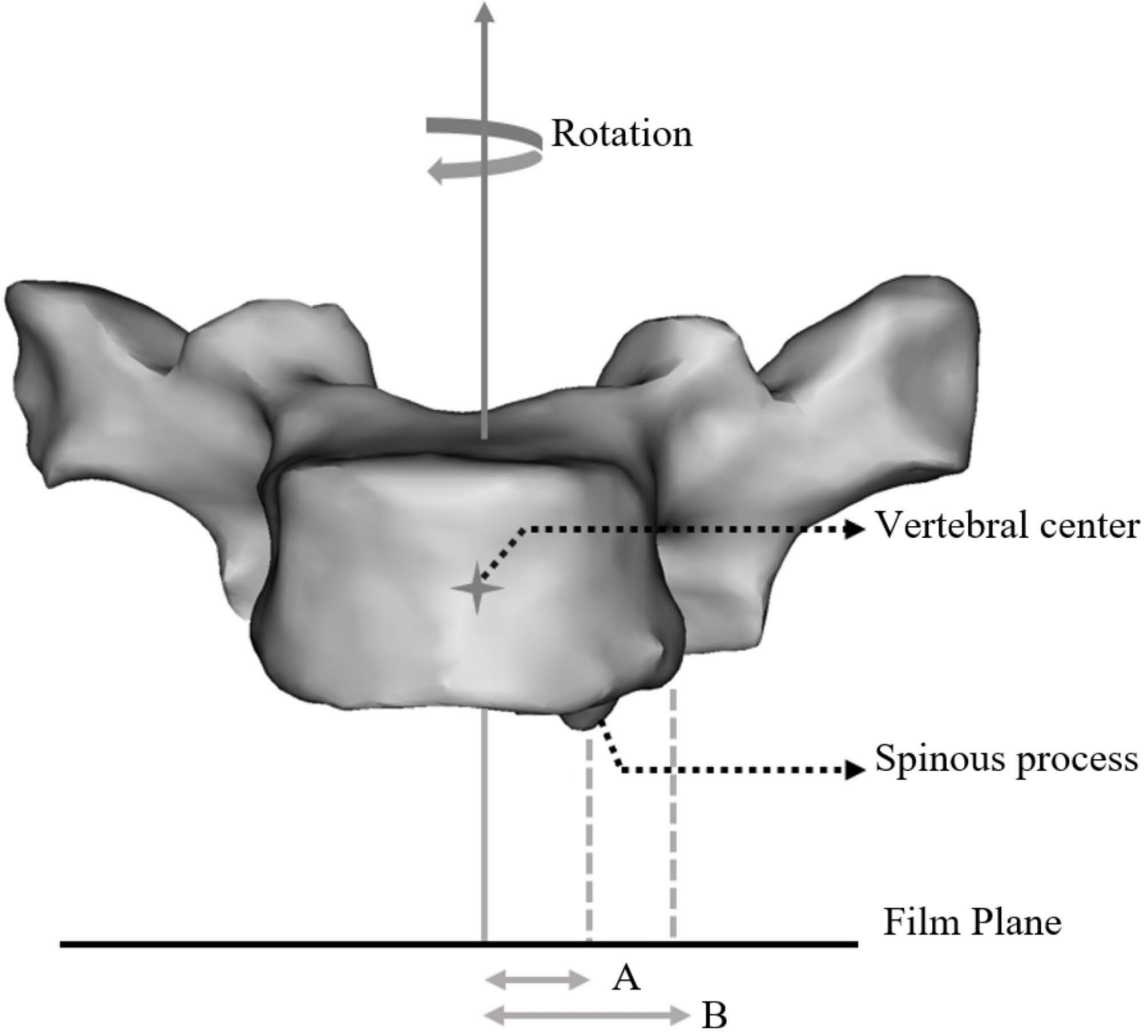
The projected distances of both spinous process and vertebral body edge from the vertebral center (A and B) are measured from the radiographic film. The displacement of the spinous process divided by the half of transverse diameter of the vertebra determine the vertebral rotation. Vertebral rotation ratio = A/B.

In reference to the Cobb method to measure vertebral rotation [5], we developed a new method to evaluate vertebral rotation. In the standing posteroanterior radiographs, the vertebral rotation ratio (VR) was calculated as the displacement of the spinous process divided by half of the transverse diameter of the vertebra. As shown in Fig. 1, the VR formula is as follows: VR = A/B.

Surgical factors were also considered. The upper end vertebra of PTC in each group was recorded. The measurement of the upper instrumented vertebra tilt (UIV tilt) was consistent with the T1 tilt. The two regions, namely, the PTC and MTC, were examined separately for implant density. Implant density was determined by dividing the number of implanted pedicle screws by the number of vertebrae involved. We applied the definition of Sato et al. regarding selective fusion [6]. Patients with lower instrumented vertebrae (LIVs) equal to or proximal to the last touching vertebra (LTV) were considered to have selective thoracic fusion (STF-LTV), and those with LIVs distal to the LTV were considered to have non-STF fusion according to LTV (non-STF-LTV). Similarly, patients were classified as STF-SV and non-STF-SV if the stable vertebra (SV) was used as the reference to evaluate the position of LIV. Using the standing film, the correction rates of PTC and MTC were calculated as the preoperative Cobb angle minus the postoperative Cobb angle divided by the preoperative Cobb angle.

### SRS-30 questionnaire

Health-related quality of life (HRQoL) outcome questionnaire, Scoliosis Research Society (SRS)-30, had been completed by these patients at the last follow-up. The 30 questions were divided into five categories: function/activity, pain, self-image/appearance, mental health, and satisfaction. The responses were graded on a 5-point Likert scale that indicated how satisfied people were with their quality of life (between favourable and unfavourable opinions). The SRS suggested scheme was used to calculate domain scores. Domain scores are calculated using a 5-point scale (5 = best outcome, 1 = poorest outcome).

### Surgical technique

All surgeries were performed by the same surgical team. The fusion area covered the structural proximal thoracic/thoracic scoliosis. In order to reduce the patient’s economic burden, we chose skip pedicle screw fixation [7, 8]. Generally, the fusion started at T2 and stopped at stable vertebrae or last touch vertebra. Pedicle screws were inserted bilaterally into the upper and lower ends of the instrumentation region. The apical regions of the two thoracic curves ensure screw placement to improve orthopedic efficiency. In other areas, we spaced a vertebra between each pair of screws. Smith-Peterson osteotomy was performed on all segments except that the two vertebrae were preserved at the proximal and distal ends of the fusion area, respectively. Several surgical maneuvers were utilized in the operation, including rod-rotation, apical vertebral derotation (by vertebral column manipulation or vertebral coplanar alignment appliance). The Legacy or CD Horizon M8 screw-rod system (Medtronic Sofamor Danek USA, Inc.Memphis, TN) were used for fixation in these patients.

### Statistical analysis

Parametric continuous variables are expressed as the means ± standard deviation. Statistical analysis was performed using SPSS 23.0 (SPSS Inc., Chicago, Illinois). According to whether these data were normally distributed, the unpaired Student’s t-test and Mann–Whitney U test were used to compare continuous variables. The χ2 test and Fisher’s exact test were used to compare categorical variables. A simple linear regression was used to investigate the association between collected radiographic parameters and T1 tilt. A stepwise multiple linear regression analysis was used to examine the radiographic variables that affected MSI. A p-value less than 0.05 was considered to be statistically significant. Two authors independently conducted the study twice in two weeks. The intraclass correlation coefficients (ICCs) were used to evaluate interobserver and intraobserver reliability, yielding values between 0.914 and 0.958 for the main radiological parameters (RSH, T1 tilt, UIVt, PTC, PTC-bending, MTC, MTC-bending and T2-VR).

## Results

### General data of patients

There were 120 consecutive patients who met the inclusion criteria and were analyzed in this study, including 32 (26.7%) males and 88 (73.3%) females, with a mean age at surgery of 15.07 ± 2.07 years (range: 11–20 years) and a mean follow-up of 30.93 ± 9.17 months (range: 24–81 months). Immediate postoperative LSI was observed in 32 (26.7%) patients, and all patients achieved balance at follow-up. For medial shoulder, preoperative MSB was observed in 16 (13.3%) patients and 36 (30%) patients immediately achieved balance. Only 37 (30.8%) patients achieved balance at follow-up. This meant that up to 69.2% of patients suffered from MSI with LSB after Lenke Type 2 AIS corrective surgery.

### Comparison among the groups

For baseline data (Table 1), the MSB group had significantly lower T1 tilt, UIVt, PTC, PTC/MTC, PTC bending, T2-VR and PTCA-VR than the MSI group (all P < 0.05). There was no significant difference in the value of age, sex, Risser Sign, RSH, MTC, TLC, C7PL-SCVL, PTCA-VT, MTC-AVT, MTC bending, flexibility of each curve or T1-VR.

**Table 1:** Preoperative comparison between MSB and MSI group

Variables	MSB group(n = 37)	MSI group(n = 83)	P
Age(years-old)	14.92 ± 2.06	15.13 ± 2.08	0.695
Sex(man/woman)	9/28	23/60	0.698
Risser Sign	3.49 ± 1.56	3.45 ± 1.44	0.705
RSH(mm)	-8.93 ± 12.49	-7.36 ± 12.21	0.625
T1 tilt(°)	1.57 ± 8.21	7.68 ± 6.36	**< 0.001**
CA(°)	2.13 ± 4.71	3.81 ± 4.44	0.131
UIVt(°)	0.87 ± 10.34	6.38 ± 7.69	**0.019**
PTC(°)	38.86 ± 6.31	43.51 ± 7.89	**0.002**
MTC(°)	61.10 ± 10.06	59.48 ± 9.92	0.413
PTC/MTC	0.65 ± 0.15	0.75 ± 0.17	**0.005**
TLC(°)	31.79 ± 8.76	29.34 ± 8.39	0.149
C7PL-SCVL(mm)	-3.92 ± 10.52	1.19 ± 15.89	0.225
PTC-AVT (mm)	4.67 ± 6.94	7.67 ± 7.25	0.061
MTC-AVT(mm)	44.93 ± 18.45	42.12 ± 16.97	0.416
PTC-bending(°)	29.58 ± 6.98	34.15 ± 7.00	**< 0.001**
MTC-bending(°)	41.45 ± 13.06	39.49 ± 11.73	0.416
TLC-bending(°)	9.06 ± 6.07	6.42 ± 8.18	0.081
Flexibility of PTC(%)	23.38 ± 9.32	21.02 ± 10.11	0.150
Flexibility of MTC(%)	33.14 ± 14.84	35.10 ± 15.57	0.416
Flexibility of TLC(%)	71.73 ± 17.89	80.37 ± 28.96	0.160
T1-VR	0.31 ± 0.23	0.36 ± 0.25	0.458
T2-VR	0.45 ± 0.27	0.58 ± 0.27	**0.003**
PTCA-VR	0.58 ± 0.24	0.75 ± 0.18	**< 0.001**

RSH: Radiographic shoulder balance; CA: Cervical axis; UIVt: upper instrumented vertebra tilt; PTC: proximal thoracic curves; MTC: main thoracic curves; PTC/MTC: the ratio of PTC and MTC; TLC: thoracolumbar/lumbar curve; C7PL-SCVL: The distance from the C7 plumb line to the midline of the sacrum; PTC-AVT: the horizontal distance between the C7 plumb line and the proximal thoracic apical vertebra. MTC-AVT: MTC-AVT: the horizontal distance between the C7 plumb line and the main thoracic apical vertebra. T1-VR: vertebra rotation ratio of T1; T2-VR: vertebra rotation ratio of T2; PTCA-VR: vertebra rotation ratio of the proximal thoracic apical vertebra

There were significant differences between the MSB group and MSI group with regard to the immediate and follow-up postoperative UIVt, PTC, PTC/MTC, PTC corrective rate, PTC-AVT, T2-VR and PTCA-VR. No significant difference existed in the values of RSH, MTC, MTC corrective rate, TLC, C7PL-SCVL, MTC-AVT or T1-VR after surgery (Tables 2 and 3).

**Table 2:** Postoperative comparison between MSB and MSI group

Variables	MSB group(n = 37)	MSI group(n = 83)	P
RSH(mm)	4.13 ± 5.72	5.65 ± 8.45	0.142
T1 tilt(°)	1.36 ± 1.96	8.48 ± 3.83	**< 0.001**
CA(°)	2.48 ± 2.70	3.68 ± 4.06	**0.029**
UIVt(°)	-0.26 ± 2.70	5.53 ± 3.54	**< 0.001**
PTC(°)	8.79 ± 3.49	18.26 ± 6.87	**< 0.001**
MTC(°)	12.05 ± 4.53	12.57 ± 6.27	0.964
PTC/MTC	0.79 ± 0.28	1.72 ± 0.96	**< 0.001**
Corrective rate of PTC	0.76 ± 0.11	0.57 ± 0.15	**< 0.001**
Corrective rate of MTC	0.80 ± 0.06	0.79 ± 0.10	0.230
TLC(°)	10.28 ± 7.72	9.64 ± 6.95	0.695
C7PL-SCVL(mm)	-8.22 ± 11.73	-4.73 ± 14.62	0.248
PTC-AVT (mm)	1.34 ± 4.46	5.94 ± 5.57	**0.001**
MTC-AVT(mm)	10.95 ± 11.27	7.43 ± 10.62	0.172
T1-VR	0.29 ± 0.16	0.33 ± 0.18	0.232
T2-VR	0.34 ± 0.17	0.55 ± 0.19	**< 0.001**
PTCA-VR	0.36 ± 0.19	0.62 ± 0.21	**< 0.001**

**Table 3:** Last follow-up comparison between MSB and MSI group

Variables	MSB group(n = 37)	MSI group(n = 83)	P
RSH(mm)	1.71 ± 5.58	2.17 ± 6.21	0.360
T1 tilt(°)	0.99 ± 1.65	8.39 ± 4.18	**< 0.001**
CA(°)	1.25 ± 2.71	3.48 ± 4.03	**0.001**
UIVt(°)	-0.16 ± 2.57	5.39 ± 3.80	**< 0.001**
PTC(°)	9.89 ± 2.57	18.85 ± 6.19	**< 0.001**
MTC(°)	13.69 ± 4.23	13.26 ± 5.83	0.405
PTC/MTC	0.77 ± 0.27	1.66 ± 0.92	**< 0.001**
Corrective rate of PTC	0.74 ± 0.09	0.56 ± 0.13	**< 0.001**
Corrective rate of MTC	0.78 ± 0.13	0.77 ± 0.09	0.603
TLC(°)	8.61 ± 7.93	10.30 ± 6.54	0.225
C7PL-SCVL(mm)	-7.19 ± 11.33	-5.42 ± 10.06	0.395
PTC-AVT(mm)	1.40 ± 3.88	6.12 ± 5.25	**< 0.001**
MTC-AVT(mm)	10.60 ± 9.29	9.11 ± 9.73	0.616
T1-VR	0.31 ± 0.16	0.35 ± 0.27	0.668
T2-VR	0.34 ± 0.19	0.60 ± 0.19	**< 0.001**
PTCA-VR	0.37 ± 0.20	0.63 ± 0.20	**< 0.001**

There was no significant difference between the MSB group and MSI group with regard to surgical factors, including the location of PTC-UEV, STF-LTV or non-STF-LTV, STF-SV or non-STF-SV and implant density of PTC or MTC (Table 4).

**Table 4:** Surgical factors for Postoperative Shoulder Imbalance

Surgical factors	MSB(n = 37)	MSI(n = 83)	P
PTC-UEV T1	31	68	0.805
T2	6	15	
LTV-LIV STF-LTV	14	28	0.663
non-STF-LTV	23	55	
SV-LIV STF-SV	21	57	0.206
non-STF-SV	16	26	
Implant Density of PTC	0.96 ± 0.32	0.95 ± 0.22	0.914
Implant Density of MTC	1.11 ± 0.29	1.08 ± 0.21	0.941

### Univariate Predictor of MSI

**Fig. 2:**
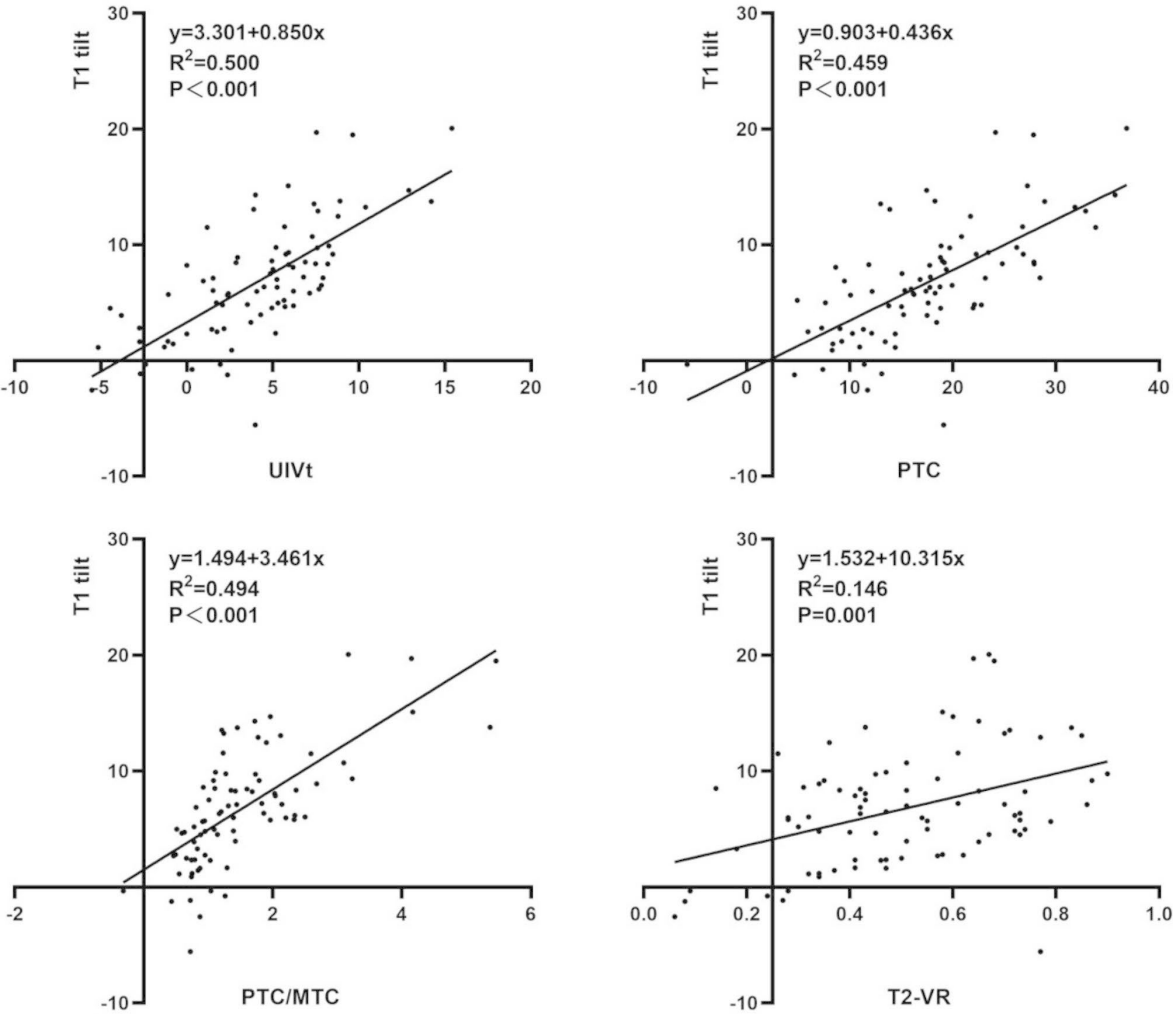
The scatter diagrams of T1 tilt at last follow-up and postoperative T2-VR, T1 tilt and PTC/MTC, T1 tilt and UIVt, T1 tilt and PTC. T2-VR, the vertebra deviation ratio of postoperative T2 vertebrae; PTC/MTC, the ratio of postoperative PTC and MTC; UIVt, UIV tilt angle

Simple linear regression analysis showed that preoperative T1tilt, UIVt, PTC, PTC bending, PTC/MTC, PTCA-VR and postoperative UIVt, PTC, PTC-AVT, PTC/MTC, T2-VR, PTCA-VR were significantly correlated with the presence of MSI. The relevant statistical results are shown in Table 5. Several example scatter diagrams are shown in Fig. 2.

**Table 5:** Correlation Coefficient Between MSB and Radiological Parameters

MSB (T1 tilt at last follow-up)	R(Pearson)	P
Preoperative T1 tilt	0.441	**< 0.001**
Preoperative UIVt	0.326	**< 0.001**
Preoperative PTC	0.375	**< 0.001**
Preoperative PTC-bending	0.418	**< 0.001**
Preoperative PTC/MTC	0.317	**< 0.001**
Preoperative T2-VR	0.138	0.132
Preoperative PTCA-VR	0.374	**< 0.001**
Postoperative UIVt	0.703	**< 0.001**
Postoperative PTC	0.656	**< 0.001**
Postoperative PTC-AV	0.478	**< 0.001**
Postoperative PTC/MTC	0.691	**< 0.001**
Postoperative T2-VR	0.413	**< 0.001**
Postoperative PTCA-VR	0.411	**< 0.001**

### Multivariate predictor of MSI

A stepwise multiple regression analysis showed that postoperative UIVt, PTC, PTC/MTC and T2-VR were significant predictors for MSI (UIVt: b = 0.398, p < 0.001; PTC/MTC: b = 2.085, p < 0.001; PTC: b = 0.155, p < 0.001; T2-VR: b = 3.536, p = 0.008; adjusted R^2^ = 0.711; Table 6).

**Table 6:** Multivariate linear regression analysis for the influence of predictors on MSI

Predictors on MSI	Unstandardized coefficient	SE	Standardized Coefficients	95% Confidence interval Lower Limit	Upper Limit	t	p
Postoperative UIVt	0.398	0.072	0.342	0.255	0.541	5.522	**< 0.001**
Postoperative PTC	0.155	0.043	0.232	0.070	0.240	3.617	**< 0.001**
Postoperative PTC/MTC	2.085	0.326	0.385	1.439	2.731	6.391	**< 0.001**
Postoperative T2-VR	3.536	1.302	0.151	0.956	6.115	2.715	**0.008**
Constant	-2.467	0.727		-3.906	-1.027	-3.395	**0.001**

### SRS-30 Scores

At the last follow-up, 72 participants completed the questionnaire. Compared the MSI group, the MSB group scored the higher in overall SRS-30 scores especially in self-image domain (4.18 ± 0.43 vs. 3.70 ± 0.35, P < 0.001), satisfaction domain (4.39 ± 0.54 vs. 3.95 ± 0.46, P = 0.001) and total average (4.31 ± 0.23 vs. 4.11 ± 0.19, P < 0.001) (Table 7). Otherwise, no significant difference was detected for comparisons among function/activity domain, pain domain and mental health domain.

**Table 7:** Last follow-up quality-of-life assessments from the SRS-30 patient questionnaire (n = 72)

Domain	MSB group(n = 25)	MSI group(n = 47)	P
Function/activity	4.22 ± 0.30	4.20 ± 0.35	0.784
Pain	4.59 ± 0.26	4.60 ± 0.27	0.934
Self-image/appearance	4.18 ± 0.43	3.70 ± 0.35	**< 0.001**
Mental health	4.18 ± 0.43	4.11 ± 0.46	0.540
Satisfaction	4.39 ± 0.54	3.95 ± 0.46	**0.001**
Total Average	4.31 ± 0.23	4.11 ± 0.19	**< 0.001**

## Discussion

Achieving good shoulder balance is one of the most significant aspects of scoliosis corrective surgery. Previous studies have mainly focused on the lateral shoulder balance [9]. However, it is not sufficient to evaluate the lateral shoulder alone. Although the lateral shoulder balance were achieved after Lenke 2 AIS corrective surgery, this study found that up to 69.2% of patients suffered from MSI. A stepwise multiple regression analysis showed that postoperative UIVt, PTC, PTC/MTC and T2-VR were risk factors for MSI. The results of the SRS-30 questionnaire were similar with those of earlier research [10], and the MSI group were dissatisfied with their appearances. The results of this study provide a wake-up call to surgeons to consider the common occurrence of MSI, and surgeons should make adequate preoperative planning to reduce the occurrence of this phenomenon.

**Fig. 3:**
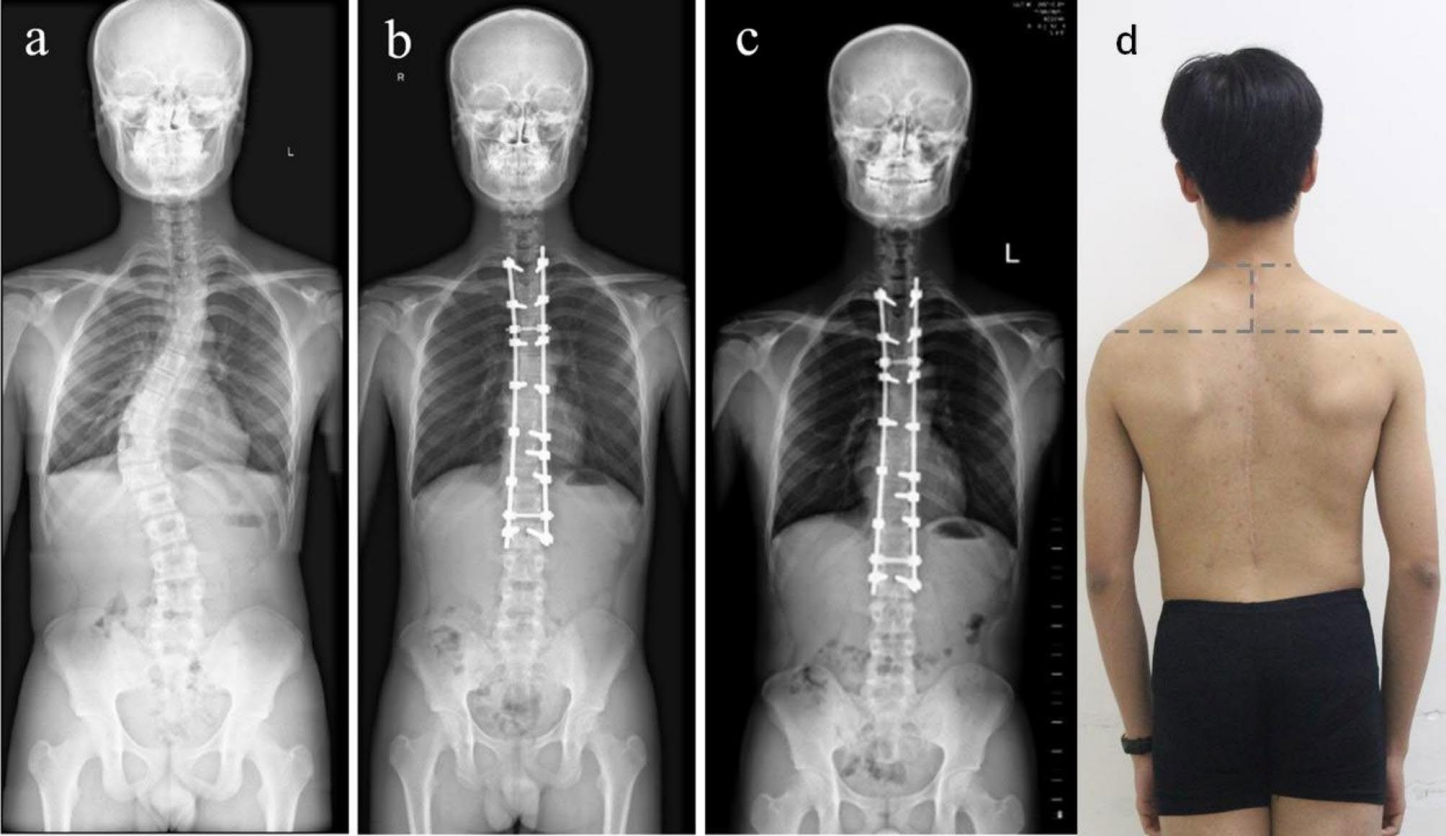
a-d. A 15 years old male patient with Lenke2 AIS fused to T2 level at the time of surgery (a-d). The RSH changed from 5.1 mm (a) preoperatively, to 0 mm at the last follow-up (c). The T1 tilt corrected from 8.2° preoperatively, to -0.8° at the last follow-up. Postoperative PTC:7.4°;UIVt:0.3°;PTC/MTC:1.26;T2-VR:0.09(b). This patient had both good shoulder balance(d)

**Fig. 4:**
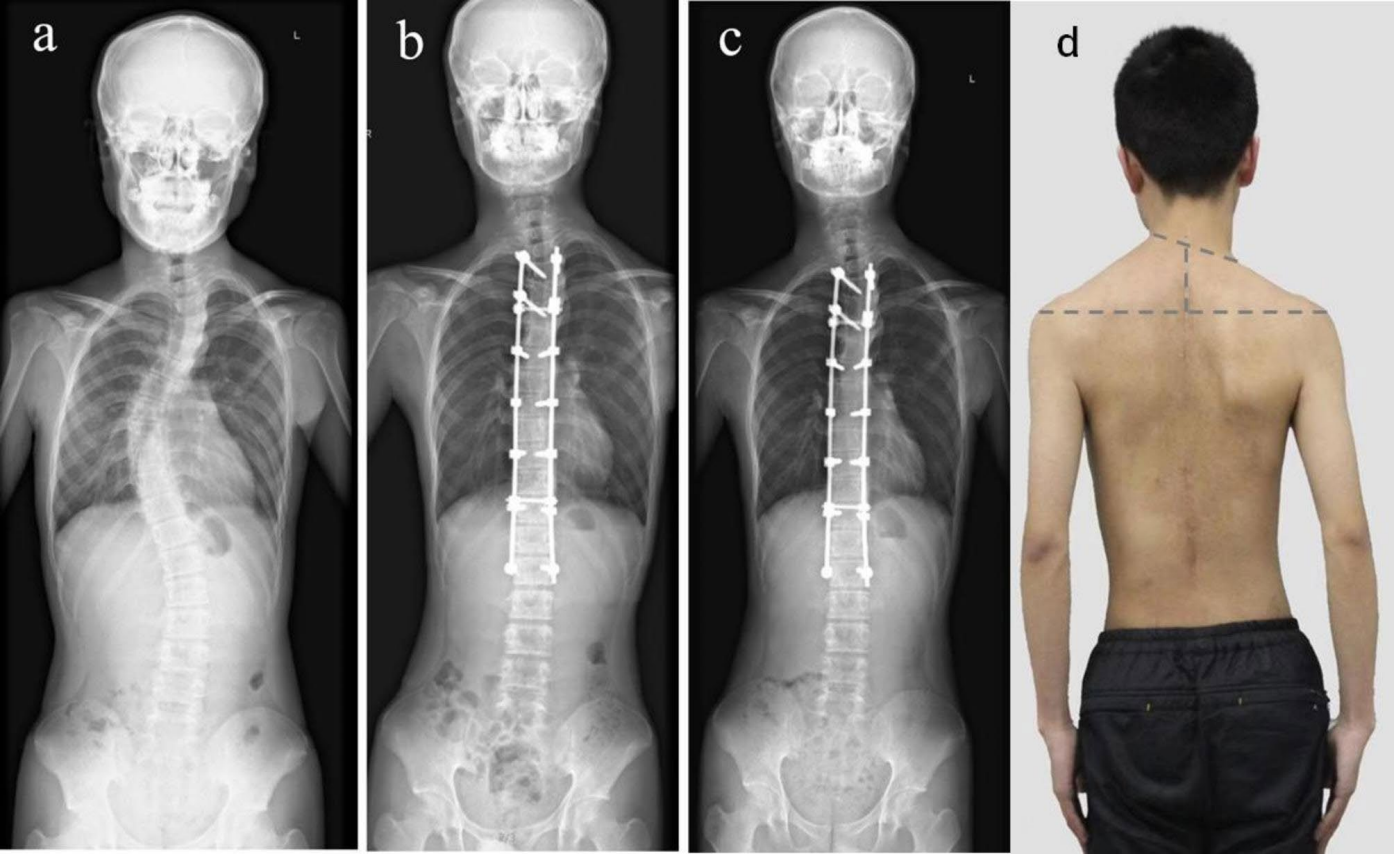
a-d. A 16 years old male patient with Lenke2 AIS fused to T2 level at the time of surgery (a-d). The RSH changed from − 6.8 mm (a) preoperatively, to 6.2 mm at the last follow-up (c). The T1 tilt changed from 15.8° preoperatively(a), to 15.1° at the last follow-up (c). Postoperative PTC: 27.22°; UIVt : 5.89°; PTC/MTC : 4.17; T2-VR:0.44 (b). This patient had MSI after surgery(d)

There is no consensus on the definition of shoulder imbalance. Lateral shoulder imbalance corresponded to RSH, Cla-A, coracoid height difference, and clavicle-rib intersection difference [11], and RSH ≥ 10 or 20 mm was usually used as the criterion of imbalance. Medial shoulder imbalance correlated well with the radiographical measurements of T1 tilt, first rib angle, and upper thoracic curve size, and a T1 tilt value greater than 3° or 4° was defined as imbalance. However, these two phenomena were independent of each other [12]. Chung et al. studied the incidence and patterns of medial and lateral shoulder discordance among 151 Lenke 1 and Lenke 2 AIS patients and found that 46.4% of AIS patients had a shoulder discordant pattern, among which Lenke 2 was as high as 67.5% [4]. In this study, we defined lateral and medial shoulder balance as RSH < 10 mm and T1 tilt < 3°, respectively. Our results with a high incidence of MSI seems to be contrary to Ono and Amir’s view that surgeons had more control correcting medial shoulder balance than clinical shoulder balance after spinal fusion and instrumentation for AIS [1, 13]. We speculated that there were three reasons for such a high incidence. First, the upper thoracic curve of Lenke 2 AIS was structural and hard to correct, making it difficult to eliminate risk factors of MSI. Second, the movement of the shoulder and sternoclavicular joints, as well as the surrounding soft tissues, might constitute a compensating mechanism for the imbalance of the lateral shoulder, which is absent in the medial shoulder. Third and most importantly, surgeons had an inadequate understanding of MSI. Through this research, we advocate that PTC should be sufficiently corrected, UIV should ensure good tilt and derotation, and PTC and MTC should be a good match to achieve postoperative medial shoulder balance. This statement needs to be confirmed in prospective studies. Typical cases are shown in Figs. 3 and 4.

Optimization of UIV tilt for postoperative medial shoulder imbalance has been widely discussed. Kwan et al. studied the relationship between postoperative UIV tilt and shoulder balance in 60 patients with Lenke1 and Lenke2 AIS and found that patients whose UIV tilt angle deviated away from the reserve motion of the UIV would have an increased risk of clinical medial shoulder imbalance postoperatively [14]. They further defined the magnitude of the value of the UIV and concluded that patients with a positive value of postoperative UIV tilt had 14.9 times increased odds of developing positive medial shoulder imbalance [15]. The results of this study also supported the results of these previous studies in which there was a significant correlation between postoperative UIV tilt and postoperative medial shoulder and neck imbalance [14–16]. In the univariate linear regression relationship between UIV and T1 tilt (T1 tilt = 0.82*UIVt + 3.05), if T1 tilt was < 3, the value of UIV was negative, which coincided with the research of Kwan et al. The understanding behind these results was that the UIV tilt tends to be positive in most patients with left upper thoracic curvature, and as a base for T1, UIV is level or negative to correct the presence of malformations.

Sufficient correction of the PTC could be associated with improved medial shoulder balance postoperatively [13]. Our results are consistent with previous studies. In the study, the preoperative PTC of the MSB group was smaller than that of the MSI group, and there was no significant difference in the flexibility of PTC and surgical factors (such as the position relationship between UEV of PTC and UIV and implant density of PTC). However, the MSB group achieved a greater PTC correction rate and a smaller postoperative PTC. This may be due to artificial surgical factors caused by different surgeons or a lack of guidance from a surgeon causing the side bending of the PTC to not be complete during the radiograph. Furthermore, several studies have reported a coordinating correction of both the PTC and the MTC consistent with postoperative LSB [17, 18]. The postoperative PTC/MTC ratio correlated with LSB [19]. In this study, we found that the postoperative PTC/MTC ratio might also be an important factor for the onset of MSI. In the report of Li et al., the shoulder height difference greater than 10 mm was divided into a PSI group and a non-PSI group, where the mean values of PTC/MTC were 1.81 and 1.56, respectively [19]. In this study, the overall mean PTC/MTC was 1.45, with 0.79 and 1.72 in the MSB and MSI groups, respectively. This meant that the balance of the medial shoulder was more rigorous for PTC/MTC than for the lateral shoulder.

Vertebral rotational deformity and decreased rotational stability may explain the pathogenesis of scoliosis and its progression [20, 21]. The pattern of vertebral rotation generally determines the curve pattern [22], and vertebral derotation in AIS can achieve promising corrections in the coronal and sagittal planes [23–25]. There are many radiographic and CT methods for vertebral rotation measurement [26]. One of these methods, called the Cobb method, divides the vertebral body into six parts, with the area where the spinous process is aligned determining the assigned grading [6]. According to this method, we further quantified it, dividing the displacement of the spinous process by half of the transverse diameter of the vertebral body to evaluate the rotation of the vertebra. Then, the T1, T2, and upper thoracic apex vertebrae were measured, and we found that their values were lower in the MSB group than in the MSI group. Multiple regression analysis showed that T2 rotation was a risk factor for T1 tilt. Therefore, we think that derotation of the upper thoracic spine can eliminate part of the cause of T1 tilt and achieve medial shoulder balance.

The strength of this study was the analysis of unique cases of LSB with MSI. At the same time, surgeons can gain a new and deep understanding of the occurrence of MSI. Nevertheless, this study has some limitations. Firstly, this is a retrospective study involving a relatively small sample size. Secondly, this study is based on radiological indicators, which cannot completely substitute for clinical parameters. Thirdly, only 60% of patients responded to the questionnaire survey. The answers of the preoperative questionnaire were incomplete and could not be included.

## Conclusion

Although the patients with Lenke 2 AIS achieve LSB after corrective surgery, up to 69.2% of them suffered from MSI. Postoperative UIVt, PTC, PTC/MTC and T2-VR were significant predictors for MSI. Sufficient correction of these variables may facilitate the achievement of MSB.

## Data Availability

Data will be available upon request to the first author Zhipeng Deng.

## Abbreviations

AIS: Adolescent idiopathic scoliosis
C7PL-SCVL: The distance from the C7 plumb line to the midline of the sacrum
LSB: lateral shoulder balance
LSI: Lateral shoulder imbalance
MSI: Medial shoulder imbalance
MSB: Medial shoulder balance
MTC: main thoracic curves
MTC-AVT: the apical vertebral translation of main thoracic curve
PSI: Postoperative shoulder imbalance
PTC: proximal thoracic curves
PTC/MTC: the ratio of PTC and MTC
PTC-AVT: the apical vertebral translation of proximal thoracic curve
PTCA-VR: the apical vertebra rotation ratio of proximal thoracic curve
RSH: Radiographic shoulder balance
TLC: thoracolumbar/lumbar curve
T1-VR: vertebra rotation ratio of T1
T2-VR: vertebra rotation ratio of T2
UIVt: upper instrumented vertebra tilt
